# Detection of Cadherin 12 in Plasma and Peritoneal Fluid Among Women with Endometriosis Using Novel Surface Plasmon Resonance Imaging (SPRi) Method

**DOI:** 10.3390/diagnostics15111366

**Published:** 2025-05-28

**Authors:** Ksawery Goławski, Zuzanna Zielińska, Cezary Wojtyła, Łukasz Ołdak, Mariusz Kuźmicki, Sławomir Ławicki, Michał Ciebiera, Tadeusz Issat, Ewa Gorodkiewicz, Piotr Pierzyński, Piotr Laudański

**Affiliations:** 1 Department of Obstetrics and Perinatology, National Medical Institute of the Ministry of the Interior and Administration, 02-507 Warsaw, Poland; 2 Doctoral School, Medical University of Warsaw, 02-091 Warsaw, Poland; 3 Club 35, Polish Society of Gynecologists and Obstetricians, 02-677 Warsaw, Poland; 4 Bioanalysis Laboratory, Faculty of Chemistry, University of Bialystok, 15-245 Bialystok, Poland; z.zielinska@uwb.edu.pl (Z.Z.); l.oldak@uwb.edu.pl (Ł.O.); ewka@uwb.edu.pl (E.G.); 5 Faculty of Health Sciences, Calisia University, 62-800 Kalisz, Poland; cezary.wojtyla@gmail.com; 6 Department of Gynecology and Gynecological Oncology, Medical University of Bialystok, 15-276 Bialystok, Poland; mariusz.kuzmicki@umb.edu.pl; 7 Department of Population Medicine and Lifestyle Diseases Prevention, Medical University of Bialystok, 15-269 Bialystok, Poland; slawomir.lawicki@umb.edu.pl; 8 Second Department of Obstetrics and Gynecology, Center of Postgraduate Medical Education, 00-189 Warsaw, Poland; michal.ciebiera@gmail.com; 9 Warsaw Institute of Women’s Health, 00-189 Warsaw, Poland; 10 Development and Research Center of Non-Invasive Therapies, Pro-Familia Hospital, 35-302 Rzeszow, Poland; 11 Department of Obstetrics and Gynecology, Institute of Mother and Child in Warsaw, 01-211 Warsaw, Poland; tadeusz.issat@imid.med.pl; 12 OVIklinika Infertility Center, 01-377 Warsaw, Poland; piotr.laudanski@wum.edu.pl; 13 Department of Obstetrics, Gynecology and Gynecological Oncology, Medical University of Warsaw, 03-242 Warsaw, Poland; 14 Women’s Health Research Institute, Calisia University, 62-800 Kalisz, Poland

**Keywords:** endometriosis, infertility, adhesion molecules, cadherin 12, CDH12, n-cadherin, biomarker, SPRi biosensor, plasma, peritoneal fluid

## Abstract

**Background:** Endometriosis is a common gynecological disease linked to significant diagnostic challenges. Cadherin 12 (CDH12), as a member of adhesion molecules, is supposed to be involved in the pathogenesis of this disease and therefore can be a potential biomarker candidate. **Methods:** In this study, we analyzed the concentration of CDH12 in plasma and peritoneal fluid samples collected from women with endometriosis and controls, using surface plasmon resonance imaging (SPRi). We collected plasma samples from 96 women and peritoneal fluid from 73 women after laparoscopy due to symptoms/ultrasound findings suggestive of endometriosis. The diagnosis was confirmed histologically. In the collected samples, we measured the concentrations of CDH12 using a novel technique utilizing an SPRi biosensor. **Results:** We found that peritoneal fluid CDH12 concentrations were lower in women with infertility compared to fertile women. However, we observed no differences in concentration of CDH12 between endometriosis and control groups in both plasma and peritoneal fluid. Additionally, in a study group of patients with confirmed endometriosis, we observed a significant positive correlation of CDH12 concentrations with patients’ age. Overall, plasma concentrations of CDH12 were significantly greater as compared to levels found in peritoneal fluid. **Conclusion:** Cadherin 12 has not been confirmed to show direct diagnostic potential for endometriosis using the SPRi method, at least in our cohort of patients.

## 1. Introduction

Endometriosis is a common and chronic inflammatory gynecologic condition affecting up to 1 out of 10 women of reproductive age and up to half of infertile women [1]. It is a chronic systemic condition, defined as multidimensional, estrogen-dependent, benign inflammatory disorder of unknown etiology. A wide range of disease extent (deep infiltrating endometriosis, ovarian endometrioma, peritoneal endometriosis, adhesions) leads to a diverse spectrum of clinical issues, including chronic pelvic pain, dysmenorrhea, dyspareunia, or infertility [2].

Awareness and interest in endometriosis are constantly growing: 2157 articles with the phrase “endometriosis” in the title were published in 2023 in PubMed in comparison to 1512 in 2018. Endometriosis is becoming a public health challenge, recently receiving increasing recognition. However, the nonspecific nature of symptoms often leads to a diagnostic delay of up to 12 years [3]. While most recent studies confirm previous European Society of Human Reproduction and Embryology (ESHRE) recommendations, the most significant change in current clinical practice is the evolution in the diagnostic process. Previously, laparoscopy was regarded as the diagnostic gold standard; it is now only recommended in patients with negative imaging results and/or where empirical treatment was unsuccessful [4]. Regardless of growing interest resulting in numerous studies and deeper research, the mechanisms behind how endometriosis originates are still unclear.

Retrograde menstruation—described by Sampson in the 1920s—is still the main theory standing behind this condition [5]. Despite the fact that nearly 90% of menstruating women have retrograde menstruation, only about 10% develop endometriosis [6]. One of the latest widely discussed theories suggests an abnormal inflammatory response to the uterine endometrium located in the pelvis as a result of retrograde transtubal flow [7]. The overreactive uterine endometrium may result in adhesion, proliferation, and implantation of ectopic tissue into peritoneal cells [8].

Studies regarding endometriosis pathogenesis have shown numerous pro- and anti-inflammatory factors, such as metalloproteinases, growth factors, cytokines, chemokines, miRNAs, and adhesion molecules, to be differentially expressed in different biological materials including peritoneal fluid, eutopic endometrium and plasma [9].

Cadherins are a group of Ca^2+^-dependent adhesion receptors that regulate changes in the organization of tissues, providing an interface for interactions between adjacent cells [10]. They are derived from a range of tissues, such as neural tissue (N-cadherin), epithelial tissue (cadherin E), and retinal tissue (cadherin R) [10]. One of the prominent representatives of the N-cadherin family is cadherin 12 (CDH12), which is responsible for cell–cell contact [11]. It consists of 794 amino acid subunits [12] and promotes cell migration, invasion, adhesion, and angiogenesis. It has been shown that CDH12 plays a role in cancer progression of colorectal cancer in a mechanism including epithelial–mesenchymal transition (EMT), the involvement of which in the development of endometriosis has also been established [13,14]. There are multiple potential mechanisms by which CDH12 may also impact fertility, based on examples of E-cadherins, N-cadherins, and K-cadherins that affect EMT, endometrial receptivity, implantation mechanisms, or oocyte meiosis [15,16,17].

CDH12 levels may be determined in body fluids using an enzyme-linked immunosorbent assay (ELISA). In our previous study on samples of peritoneal fluid of endometriosis patients, it showed a range between 2 and 10 ng/mL [18]. For obvious reasons related to the ease of sampling, the most desirable approach in researching the biomarker value of this molecule would be to use samples of blood plasma [19]. However, the application of ELISA and Western blotting for the determination of CDH12 in serum has not been shown to cover the desired sensitivity range [19]. Considering this, our group developed a novel technique for the detection of CDH12 that is based on the application of a biosensor-based array surface plasmon resonance imaging (SPRi) technique [19]. The method allows the determination of CDH12 and other molecules within the required concentration ranges without needing signal amplification or prior sample treatment. It offers the benefits of a linear analytical signal, the use of small sample volumes, and the ability to monitor biomolecular reactions in real time [20]. This method measures the changes in the refractive index caused by molecules bonded to the metal surface, allowing the determination of the concentration of the analyte directly in the sample [21]. It shows higher sensitivity and specificity as well as cost-effectiveness over conventional techniques [22]. It does not require signal enhancement or preliminary sample processing, resulting in the ability to use very small samples of body fluid [19]. SPRi biosensors have been increasingly applied in medical diagnosis where rapid and sensitive methods are required for the determination of substances that are potential markers of disease, and SPRi detection is believed to be the most promising among surface plasmon-based techniques [23].

The aim of this study was to measure the concentration of CDH12 in plasma and peritoneal fluid samples collected from women with and without endometriosis using the optical method of SPRi and to examine potential interrelations between the studied group and patients’ characteristics. Such an approach was also intended to verify results based on peritoneal fluid samples measured with the ELISA method in our previous study [18].

## 2. Materials and Methods

### 2.1. Materials and Reagents

The following antibodies were used in the study: monoclonal recombinant rat anti-CDH12 antibody, recombinant human CDH12 protein (each >95% pure), cysteamine hydrochloride, 1-ethyl-3-(3-dimethylaminopropyl)carbodiimide, N-hydroxysuccinimide (NHS), 0.2 M carbonate buffer pH = 8.50 Tween-20, absolute ethyl alcohol (99.8%), ethyl alcohol 96%, buffered saline (PBS) (with pH = 7.40), and immersion oil 518. The biosensor base was a 1 mm thick glass plate with a 50 nm gold layer. The detailed information can be found in Appendix A.

### 2.2. Patients’ Characteristics and Sample Collection

Samples of peritoneal fluid and blood were collected in a multicenter, cross-sectional study undertaken at eight departments of obstetrics and gynecology in Poland. The details of recruitment and precise methodology were published in previous publications from our group [18,24,25].

Study and control groups comprised regularly menstruating women aged 18–40 years who were qualified for laparoscopy due to symptoms / ultrasound findings suggestive of endometriosis (pelvic pain, idiopathic infertility, presence of endometrioid-like ovarian cysts). In the course of their laparoscopy, patients were diagnosed with endometriosis using the revised American Fertility Society classification [26], and histopathologic examination confirmed these diagnoses. All participants also completed the World Endometriosis Research Foundation (WERF) clinical questionnaire [27]. Patients with confirmed endometriosis were classified as the study group. The control group was formed from disease-free patients without visible endometriosis on their laparoscopy.

In the analysis, we included samples of plasma from 96 patients (52 in the study group and 44 controls) and peritoneal fluid from 73 patients (41 in the study group and 32 controls). Among these, both plasma and peritoneal fluid samples were available in 57 cases (31 in the study group and 26 controls).

Written informed consent was collected from all patients, and the study was approved by the Ethics Committee of Medical University of Warsaw (AKBE/133/2020). Material collection did not have any impact on the medical management of patients and was performed in accordance with the Declaration of Helsinki.

Every procedure for securing the plasma and peritoneal fluid samples was performed following the Endometriosis Phenome and Biobanking Harmonisation Project standard operating procedures [28]. Detailed information regarding the collection and structure of the study groups is presented in Appendix B.

### 2.3. SPRi Analysis

A novel method employed for the determination of CDH12 is based on a biosensor using surface plasmon resonance imaging (SPRi) detection [19]. SPRi examines plasmons, the surface electrons of metals. The excitation of plasmons depends on the refractive index of a thin layer of the medium placed on the metal, such as gold. The binding of biomolecules on the surface—in this case, the binding of an antibody specific to CDH12—causes a change in the refractive index. The analytical signal is the difference in light intensity reflected before and after interaction with the analyte. Due to the proportionality of the generated signal to the mass immobilized on the gold surface, it is possible to examine the analyte in the sample quantitatively [29]. A more detailed description of the aforementioned method can be found in Appendix A and the separate article by Oldak et al. [19]. CDH12 concentrations were read from a calibration curve prepared for the study in the 0 to 5 ng/mL range. Samples were diluted 5-fold with PBS buffer to fit within the linear range of the biosensor.

### 2.4. Data Analysis

CDH12 levels were examined between different groups of patients: patients with and without endometriosis, patients with different stages of endometriosis, and patients with and without infertility. The significance of differences between groups were, after testing for normal distribution using the Shapiro–Wilk test, evaluated with the Mann–Whitney U-test and Student’s *t*-test. To assess differences in CDH12 concentrations in two body fluids, a *t*-test for dependent groups was performed (after testing for normal distribution). Outliers were detected and then excluded using classic statistical domain based on the interquartile range. Correlations between variables were calculated using Spearman’s rank correlation coefficient. Differences between women percentages based on infertility and its types, as well as phase of the cycle, were tested with a test for two indicators of structure. The level of statistical significance was set at *p* < 0.05. Statistical analysis was performed with Statistica 13.0 (TIBCO Software, Palo Alto, CA, USA).

No formal power calculation was conducted prior to this exploratory study. Sample size was determined by the availability of high-quality material from a multicenter biobank developed by our group. With 96 plasma and 73 peritoneal fluid samples, we considered the dataset sufficient for initial analysis, particularly given the invasiveness of fluid collection.

## 3. Results

### 3.1. Peritoneal Fluid

#### 3.1.1. Clinical Characteristics of Patients

In this study, we enrolled samples of peritoneal fluid collected from 73 patients, i.e., 41 with endometriosis (study group) and 32 disease-free controls. Simultaneous collection of both peritoneal fluid and plasma samples was carried out in 57 (57/73; 78%) of the included patients (among them, 31 were in the study group and 26 were controls).

Clinical characteristics of patients in the study and control groups, including age, fertility status, and cycle phase at the time of laparoscopy, are presented in Table 1. Within the study and control patients, there were 41 patients with infertility. There were no significant differences in analyzed clinical parameters between the study and control groups.

#### 3.1.2. CDH12 Concentrations in Peritoneal Fluid

There were no significant differences in CHD12 concentrations in peritoneal fluid between the study and control groups (*p* = 0.35). The median concentration in the study group was 17.11 ng/mL whilst in controls it was 17.63 ng/mL. Similarly to our previous work [19], we also aimed to relate CDH12 concentration in peritoneal fluid to clinical factors such as fertility status, age, phase of the cycle, stage of endometriosis, and presence of ovarian cysts. Among these, we confirmed significantly lower peritoneal fluid concentrations of CDH12 in patients with infertility as compared to fertile ones (14.772 ng/mL vs. 22.179 ng/mL, *p* < 0.05) (Table 2).

### 3.2. Plasma

#### 3.2.1. Clinical Characteristics of Patients

Plasma was collected from 96 patients: 52 in the study group and 44 in controls.

As described in the previous section, 57 of these patients also had peritoneal fluid collected. Thus, plasma samples from 38 patients are original records not involved in the peritoneal fluid collection.

CDH12 concentrations did not differ significantly between groups (*p* = 0.36), but values are different (median concentration 25.15 ng/mL in the study versus 22.05 ng/mL in the control group).

We also observed no differences between groups for the remaining analyzed variables, as presented in Table 3.

#### 3.2.2. CDH12 Concentration Levels for Different Clinical Features

CDH12 plasma concentrations were analyzed in relation to factors such as fertility status, age, day and phase of cycle, stage of endometriosis, and the presence of ovarian cysts (Table 4).

Similarly to the peritoneal fluid analysis, we distinguished several groups based on the above factors. We have observed statistical significance of CDH12 concentrations only for age (*p* = 0.046).

**Figure 1:**
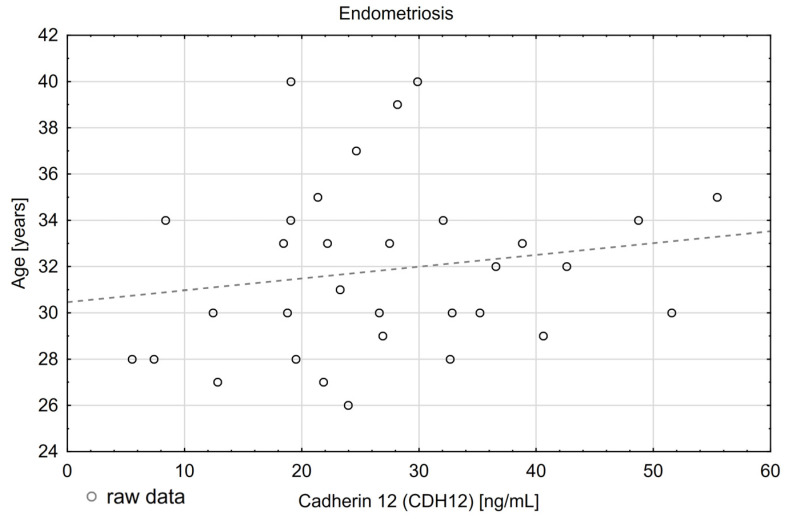
Correlation of plasma CDH12 concentrations [ng/mL] with age.

Moreover, we have noticed a weak positive correlation that was statistically significant (*p* = 0.044 r_S_ = 0.3) between age and CDH12 concentration levels in the study group (Figure 1 and Table 4). We did not observe such a phenomenon in the control group (r_S_ = 0.1; *p* = 0.573). There was a statistically significant weak positive correlation for the whole group (*p* = 0.046 r_S_ = 0.20).

**Figure 2:**
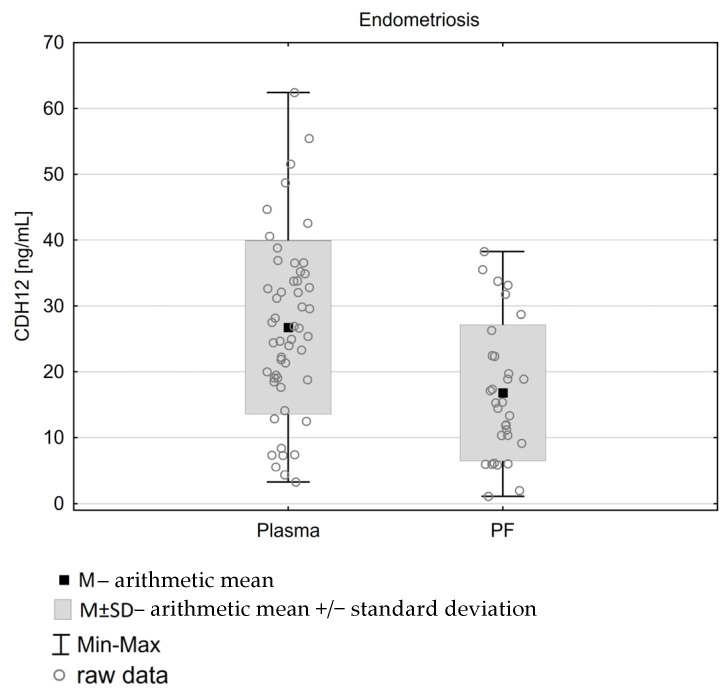
Concentrations of CDH12 [ng/mL] in plasma and peritoneal fluid.

In the study group, we found that CDH12 concentration in plasma was significantly greater than in peritoneal fluid (M ± CV; 27.23 ng/mL ± 53% vs. 16.79 ng/mL ± 62%) (Table 5, Figure 2). Such a phenomenon was not confirmed within the control group, as the comparison of CDH12 concentrations between plasma and peritoneal fluid yielded no significant differences. In turn, we found no significant correlation between the peritoneal and plasma CDH12 levels (see Table 5)

## 4. Discussion

The etiopathology of endometriosis is a subject of continuous research. One of the prominent mechanisms involves the altered function of adhesion molecules, allowing ectopic attachment of endometriotic cells [23]. One such molecule, Cadherin 12 (CDH12), has been implicated in the pathogenesis of endometriosis and infertility [14,16,30].

Previously, we described the potential role of CDH12 in the pathogenesis of endometriosis by investigating samples of peritoneal fluid from patients with endometriosis compared to controls, but there are no more examples of such articles [18]. This study aimed to elucidate on the diagnostic potential of CDH12 in patients with endometriosis and infertility.

The search for a validated method for the determination of CHD12 status directed our group toward creating a novel, optical biosensor-based method of surface plasmon resonance imaging (SPRi) detection of CDH12 in blood plasma, which we viewed as progress compared to methods based on the evaluation of peritoneal fluid [21]. SPRi offers advantages over conventional analytical techniques because its optical detection technique allows for the direct and real-time detection of many biological and chemical substances [22]. It is highly sensitive, specific, and also cost-effective [22]. This may explain why it is increasingly applied in medical diagnosis, where sensitive but also rapid methods are required [31].

Comparing the results of our previous studies, it was noticeable that CDH12 readings performed by SPRi were greater than measurements employing ELISA. We think that a potential explanation is that SPRi may show higher measurements than ELISA due to its greater sensitivity, ability to measure real-time interactions, and potential to detect weak or non-specific binding. ELISA, while specific, may underreport concentrations due to limitations in detection sensitivity, signal amplification, and the accessibility of bound molecules.

We did not find statistically significant differences between CDH12 concentrations in plasma and peritoneal fluid among patients with and without endometriosis.

This directly corresponds with the results of our previous study, which investigated CDH12 concentration levels in a similar group of patients, but only in peritoneal fluid using the ELISA method [18].

Previously, we also showed statistically significant differences in CDH12 concentrations in peritoneal fluid among patients with endometriosis and infertility compared with controls without these conditions [18]. In the current study, however, we did not find a confirmation of this for peritoneal fluid or plasma. Nevertheless, we found statistically significant differences for CDH12 peritoneal fluid concentrations between fertile and infertile women. Even considering that endometriosis might be responsible for a significant proportion of the female factor of infertility, we find this result noteworthy as it might point to some endometriosis-independent mechanisms of infertility involving CDH12 [18]. On the other hand, no such relationships were found for plasma samples in the current study.

Surprisingly, we noted higher CDH12 concentrations in plasma compared to peritoneal fluid. The potential background for such a finding was introduced by Kajdos et al., who described microvesicles (MVs) among patients with endometriosis versus controls, where the MV concentration was higher in plasma than peritoneal fluid, which can possibly be present regarding adhesion molecules [32].

An intriguing finding of this study was the statistically significant positive correlation of CDH12 concentration in plasma with patients’ age for the whole and the study group (no such correlation was found for controls). Although we cannot definitively explain this observation [33,34,35], we found examples of statistically significant positive age correlations with the concentration of molecules involved in epithelial–mesenchymal transition (a well-known process in the pathogenesis of endometriosis described earlier in this article) [23,33,34,35,36]. For instance, Singh et al. presented enhanced expression of N-cadherin correlated with stage and age among patients with urothelial carcinoma of the bladder [36,37]. E-cadherin was inversely correlated with age in patients with benign prostate hyperplasia [38]. A potential link with CDH12 (N-cadherin family member) is suggested by examples where N-cadherin expression was negatively correlated with that of E-cadherin [37]. Considering our clinical material, the significant, yet still relatively weak, correlation between CDH12 concentrations and age requires further study, especially focusing on different types of infertility. It is noticeable that CDH12 is most likely an adjunctive “chainlink” rather than a primary driver of endometriosis. Its presence might, however, be relevant in its more specific subtypes as well as types of infertility.

We recognize that this study is limited by its restricted number of observations. Yet, the sample size allowed the detection of significant associations between CDH12 levels and infertility as well as patients’ age, indicating adequate sensitivity for biologically relevant effects. Enrolling a larger number of patients in future studies would enable a more thorough investigation of these issues while maintaining statistical power. A direction for future research in this field might be an evaluation of differences in CDH12 concentrations, comparing patients diagnosed with primary infertility versus those with secondary infertility overall, as well as comparing patients with endometriosis versus those without. For now, the total number of 8 patients with collected peritoneal fluid and 13 with plasma diagnosed with secondary infertility does not give such an opportunity.

## 5. Conclusions

The search for new biomarkers of endometriosis may facilitate a more efficient diagnostic process. In our material, the results did not confirm the direct diagnostic potential of CDH12 in patients with endometriosis, despite using a novel and more sensitive method of its evaluation as well as of using samples of both plasma and peritoneal fluid. However, in the investigative process, we found some interesting relationships regarding CDH12 concentrations in relation to patients’ age and fertility status. This corresponded with our previous results, which focused only on peritoneal fluid using the ELISA method, and may require further attention in future research. Although our findings did not strongly support the potential of CDH12 as a marker for endometriosis or infertility, its biology, which is closely linked to the pathogenesis of both conditions, warrants further studies. Focusing on its determination in other biological fluids, such as endometrial fluid or menstrual blood, could be an additional route to follow, especially in infertile women and women with endometriosis, respectively. Another promising direction would be to explore CDH12 in combination with other markers.

To sum up, CDH12 might play a diagnostic role in specific clinical conditions in the future, and the relationship between CDH12 concentration and patients’ age presents an interesting avenue for future investigation.

## Figures and Tables

**Table 1:** Patient characteristics—peritoneal fluid.

Variable	Endometriosis (*n* = 41)	Controls (*n* = 32)	*p*-Value
Cadherin 12 (CDH12) [ng/mL] PF			0.35
Me ± IQR; QCV; n	17.11 ± 17.16; 100%; 41	17.63 ± 24.88; 141%; 32	
(Min–Max)	(1.08–52.70)	(2.15–48.15)	
Age [years]			0.07
M ± SD; CV; n	32.2 ± 4.4; 13.7; 40	29.9 ± 6.1; 20.3; 32	
95% PU	(30.8–33.6)	(27.7–32.1)	
Day of cycle			0.07
Me ± IQR; QCV; n	12.0 ± 9.5; 79%; 40	10.0 ± 8.0; 80%; 32	
(Min–Max)	(5.0–26.0)	(5.0–26.0)	
Infertility	25 (61%)	16 (50%)	0.35
Primary infertility	21 (51%)	12 (38%)	0.27
Secondary infertility	4 (10%)	4 (13%)	0.69
First phase of cycle	25 (63%)	23 (72%)	0.42

M—arithmetic mean; Me—median; SD—standard deviation; IQR—interquartile range; CV—Pearson’s coefficient of variation; QCV—coefficient of quartile variation (IQR/Me); ×100%.

**Table 2:** CDH12 concentration levels in peritoneal fluid samples in relation to fertility status.

Variable	Patients with Infertility	Patients Without Infertility	*p*-Value
Cadherin 12 (CDH12) [ng/mL] PF			**0.05**
Me ± IQR; QCV; n	14.772 ± 13.205; 89%; 41	22.179 ± 26.322; 119%; 32	
(Min–Max)	(1.081–42.290)	(1.964–52.700)	

M—arithmetic mean; Me—median; SD—standard deviation; IQR—interquartile range; CV—Pearson’s coefficient of variation; QCV—coefficient of quartile variation (IQR/Me); ×100%.

**Table 3:** Patient characteristics—plasma.

Variable	Endometriosis (*n* = 52)	Controls (*n* = 44)	*p*-Value
Cadherin 12 (CDH12) [ng/mL] PF			0.3561
Me ± IQR; QCV; n	25.15 ± 15.73; 62%; 52	22.05 ± 26.6; 107%; 44	
(Min–Max)	(3.25–55.46)	(2.15–67.85)	
Age [years]			0.6564
Me ± IQR; QCV; n	31.50 ± 6.50; 21%; 52	31.0 ± 7.0; 23%; 43	
(Min–Max)	(24.0–43.0)	(19.0–46.0)	
Day of cycle			0.1261
Me ± IQR; QCV; n	11.0 ± 7.5; 68%; 52	10.00 ± 6.50; 65%; 44	
(Min–Max)	(4.0–26.0)	(1.0–27.0)	
Infertility (n; %)	32; 61.54%	26; 59.09%	0.8068
Primary infertility (n; %)	26; 50.00%	19; 43.18%	0.5046
Secondary infertility (n; %)	6; 11.54%	7; 15.91%	0.5330
First phase of cycle (n; %)	16; 30.77%	10; 22.73%	0.4417

Me—median; SD—standard deviation; IQR—interquartile range; CV—Pearson’s coefficient of variation; QCV—coefficient of quartile variation (IQR/Me); ×100%.

**Table 4:** Statistical significance of CDH12 plasma concentrations depending on clinical factors.

Factor	*p*-Value
**Age (N = 96)**	0.046 *
**Age in research group (n = 52)**	0.044 *
Age in control group (n = 44)	0.573
Infertility (*N* = 38 + 58)	0.597
Primary infertility (*N* = 51 + 45)	0.186
Secondary infertility (*N* = 83 + 13)	0.243
Phase od cycle (*N* = 70 + 26)	0.141
Ovarian cysts (*N* = 68 + 28)	0.945
Stage o endometriosis (I, II vs. III, IV, *N* = 27 + 23)	0.186
Day of cycle (*N* = 96)	0.343 *

* *p* value for Spearman’s rank correlation coefficient.

**Table 5:** Concentrations of CDH12 [ng/mL] in plasma and peritoneal fluid.

		M	SD	CV	T	*p*	r_S_	*p*
**Study group** ***N* = 31**	Plasma	27.2	14.485	53%	2.90	0.007	−0.29	0.112
	Peritoneal fluid	16.79	10.331	62%				
**Control** ***N* = 26**	Plasma	24.78	17.43	70%	1.22	0.234	0.18	0.234
	Peritneal fluid	19.85	12.58	63%				

M—arithmetic mean; SD—standard deviation; CV—Pearson’s coefficient of variation; T—t-value; *p*—significance level for concentration and correlation; respectively; r_S_—Spearman’s correlation coefficient.

## Data Availability

Data are contained within the article.

## Appendix A

### Appendix A.1. Materials and Reagents

The following antibodies were used in the study: monoclonal recombinant rat anti-CDH12 antibody (catalog no. MAB2240) and recombinant human CDH12 protein (catalog no. 2240-CA), each >95% pure (R&D Systems, Minneapolis, MN, USA), cysteamine hydrochloride, 1-ethyl-3-(3-dimethylaminopropyl)carbodiimide (EDC) (Merck, Darmstadt, Germany), N-hydroxysuccinimide (NHS) (Merck, Darmstadt, Germany), 0.2 M carbonate buffer pH = 8.50 (Merck, Darmstadt, Germany) and Tween-20 (Merck, Darmstadt, Germany). Absolute ethyl alcohol (99.8%) and ethyl alcohol 96% were purchased from Pol Aura (Morąg, Poland), and buffered saline (PBS) with pH = 7.40 was purchased from Biomed (Lublin, Poland). Immersion oil 518 Immersol was purchased from Zeiss (Jena, Germany). The biosensor base was a 1 mm thick glass plate with a 50 nm gold layer provided by Ssens (Enschede, The Netherlands).

### Appendix A.2. Materials and ReagentsSPRi Analysis

A quartz crystal microbalance was used to analyze the characteristics of the formation of successive layers of the biosensor, from the linker monolayer to the final capture of CDH12 from solution [21]. The determined analytical parameters, namely the values determining the accuracy, precision, and repeatability of the method, do not exceed the permissible 20% deviations. The proposed method is also selective with respect to possible potential interferents, occurring in up to 100-fold excess concentration relative to the CDH12 concentration [21]. The determined limit of quantification (LOQ—4.92 pg mL^−1^) indicates the possibility of performing quantitative analysis in human plasma and peritoneal fluid without the need to concentrate the samples. The Passing-Bablok regression model revealed good agreement between the reference method and the SPRi biosensor, with Spearman’s rank correlation values of −0.961 and 0.925.

## Appendix B

During the procedures, peritoneal fluid was collected via Veress needle aspiration under direct visual inspection in the beginning of the laparoscopy in order to avoid contamination with blood. 10 milliliter samples of venous blood were collected on the day of and prior to the surgery.

From the study we excluded patients on any form of hormonal therapy during last 3 months before laparoscopy, as well as ones with malignant or suspected malignant disease, autoimmunological disease, previous and/or current pelvic inflammatory disease, any pelvic surgery before, uterine fibroids or polycystic ovaries.

Cycle phase was calculated based on the last menstrual period and average length of menstrual cycle. Moreover, the phases of menstrual cycle in women with and without endometriosis were determined by histological dating of eutopic endometrial samples collected simultaneously with pathological lesions.
